# Breast cancer knowledge, attitudes, and screening behaviors among African American women: the Black cosmetologists promoting health program

**DOI:** 10.1186/1471-2458-7-57

**Published:** 2007-04-17

**Authors:** Georgia R Sadler, Celine M Ko, Jennifer A Cohn, Monique White, Rai-nesha Weldon, Phillis Wu

**Affiliations:** 1 Moores UCSD Cancer Center, University of California, San Diego, 3855 Health Sciences Drive #0850, La Jolla, CA, 90293-0850, USA

## Abstract

**Background:**

African American women have higher rates of breast cancer mortality than their white counterparts. Studies have suggested that this is partly caused by discovery of cancer at a later stage, highlighting the importance of encouraging early detection of breast cancer in this population. To guide the creation of a breast cancer education intervention and help focus other health educators' and clinicians' health promotion efforts, this study explored whether a cohort of African American women living in San Diego would demonstrate the possession of adequate baseline knowledge about breast cancer screening and adherence to widely recommended screening guidelines.

**Methods:**

African American women (N = 1,055) from San Diego, California participated in a beauty salon-based survey about breast cancer knowledge, attitudes, and screening practices. Women's ages ranged from 20 to 94 years, with average age of 42.20 (SD = 13.53) years. Thirty-four percent reported completing college and/or some graduate school training, and 52% reported having some college or post high school formal training. Seventy-five percent of the sample reported working outside their home. Participating cosmetologists and their salons were recruited to the study through word-of-mouth referral by highly respected African American community leaders.

**Results:**

Salon clients reported low rates of adherence to recommended breast cancer screening guidelines. Of the 1,055 participants, 31% reporting performing breast self-exam every month. Of those participants 40 and older, 57% reported having had a clinical breast exam and 43% reported having had a mammogram in the past year. Knowledge of breast cancer was associated with adherence to screening guidelines. While women recognized the serious health threat that breast cancer poses and that early detection of breast cancer is important, only 30% of women reported feeling well informed about the disease. Many participants demonstrated a lack of basic knowledge about breast cancer. The Health Belief Model postulates that access to such information is an essential element in the progression toward engaging in screening behaviors.

**Conclusion:**

Data from this study reflect a continuing need for increased breast cancer education for African American women. In light of the considerable mainstream information available related to breast cancer, these data reinforce the need for more breast cancer education programs that are clearly intended to attract the attention of African American women.

## Background

Breast cancer is the most common malignant neoplasm and the second leading cause of cancer death among women in the United States. Even though breast cancer incidence is lower among African American women (119.4/100,000) than among Caucasian women (141.1/100,000), African American women's death rate has consistently remained higher (34.7/100,00 vs. 25.9/100,000). An estimated 19,620 new diagnoses of invasive breast cancer and 5,670 deaths from breast cancer are expected to occur among African American women in 2006 [1,2]. The adherence to recommended breast cancer screening guidelines is frequently reported to be lower in the African American population and is one of the factors that is believed to contribute to this disparity. Other factors that have been identified as having the potential to contribute to the unfavorable disparity in mortality rates among African American women include genetic differences, treatment delays, and differences in the treatments provided [3-9]. Of all of the potential contributors, adherence to recommended screening guidelines is one which can be immediately addressed with available knowledge while clinicians, public health officials, and scientists search for additional ways to address the other factors contributing to these disparities. It is widely accepted that interventions to increase adherence to breast cancer screening guidelines among African American women could contribute to a considerable reduction in the number of women with late stage diagnoses and subsequently, the breast cancer mortality rate among African American women [2].

The Health Belief Model (HBM) [10], which has been widely used to frame research studies related to the prediction of health-related behaviors in relation to health belief patterns, was selected as the frame of reference for the current study of African American women's breast cancer screening behaviors. Women's self-reported breast cancer knowledge, attitudes, behaviors, and perceptions of support, and the components of the HBM (perceived susceptibility, perceived benefits, cues to action, self-efficacy) were explored in this paper.

## Methods

This manuscript reports on baseline data about the breast cancer knowledge and screening practices among the sample of 1,055 African American women. The women, who were clients of the 20 salons of *The Black Cosmetologists Promoting Health Program*, provided written consent to participate in the program's randomized controlled education trial [11,12]. The salons, which catered to a clientele of predominantly African American women, had accepted the invitation to help evaluate the effectiveness of a breast cancer train-the-trainers intervention in which the cosmetologists would serve as the lay health educators. The salons were identified through word-of-mouth referral by highly respected African American community leaders. As a first step in defining the parameters of the educational intervention to be offered, the baseline data were collected from the salon's participating clients between January 1998 and October 1999. The data collected from those surveys are reported in this paper.

Research assistants, in collaboration with the stylists, invited the salons' clients to participate in the study. To allow clients to be recruited at times when the research assistants were not present and thereby increase the diversity of the client sample, the participating cosmetologists were also trained to recruit the salons' clients to the study and conduct the consenting process on their own. This was facilitated by placing a study display in the salon next to a large, eye-catching basket of beauty supplies that were to be raffled off at the conclusion of the study accrual. The display included clear instructions on how to complete the baseline survey and consent forms and informed the client to ask the cosmetologist if they needed more information or assistance with the documents' completion. All elements of the study were approved by the University of California, San Diego's Institutional Review Board. A more detailed description of the methodology employed is provided in previously published papers [11,12].

Of the 530 women who were approached by a research assistant, 13% (71/530) refused (When the research assistant was not present at the salon, it was not possible to make an exact calculation of the number of women who refused to participate). The 71 women who refused to participate were asked by the research assistant if they would anonymously answer four, simple, IRB-approved questions that would help the research team determine whether women who opted not to participated were comparable to women who agreed to participate in the study. All agreed to do so. When asked for their reason for not participating, 12 women reported that they did not want to spend the time, 29 refused to give a reason, and the remainder of the 71 women gave a variety of miscellaneous reasons. T-tests showed no difference in education level between the women who refused to participate versus those who participated. However there was a significant difference in age (p < .05, data not shown) between the women who participated and those who opted not to participate. Women who opted not to participate were somewhat older than those who agreed to participate.

While San Diego has 184 different postal ZIP codes, the salons that were referred to the research team by the community leaders were from 13 ZIP codes. According to the 1990 and 2000 Census, each of these ZIP codes is home to a high proportion of African Americans. Slightly more than half (52% (550/1055)) of women who participated in this study reported home addresses that were similarly clustered into these same thirteen ZIP codes, suggesting that these salons would be viewed as an integral part of the community's overall neighborhood.

## Results

### Sample description

The sample was composed of 1,055 African American women. All the participants lived in San Diego County, and all but 42 reported their ages, which ranged between 20 and 94 years of age. For those women who gave their ages, the mean age was 42.20 (SD = 13.53) years. Those with missing age data were not included in the calculation of the mean age and in any of the subsequent analyses on screening behaviors. Thirty-four percent (356/1055) of the women had completed college and/or had at least some graduate school training, half of them (52% (547/1055)) had some college or post high school formal training, and 11% (122/1055) had completed high school or less. Three percent (30/1055) did not provide information on educational level. Most of the women (75% (790/1055)) reported that they worked outside their home; among the 945 women aged 65 years and younger, 80% (756) reported working outside the home. Thirteen (1%) of the 1055 women reported they had breast cancer.

### Breast cancer knowledge

In spite of the fact that most women agreed with the statement "The more I know about a disease, the more control I have," only 30% (317/1055) of the women reported feeling that they were very well informed about breast cancer. This perceived lack of knowledge takes on greater significance because women who reported that they perceived that they were well informed about breast cancer were significantly more likely to be adhering to recommended breast cancer screening guidelines for breast self-exams, clinical breast exams, and mammograms (Table 1). However, this observation cannot distinguish between whether the sense of being well informed prompted adherence to screening guidelines or whether adherence to screening guidelines prompted a sense of being well informed about breast cancer. Further research is warranted to determine whether helping women to become more well informed about breast cancer will increase their odds of adhering to recommended screening guidelines. This is the focus of the proposed educational intervention that will follow this baseline data collection.

**Table 1:** Screening adherence by perceptions of how informed they were about breast cancer

Education level	Reported BSE within past month*		Reported CBE within past year (for women 40 + only) **		Reported Mammogram within past year (for women 40 + only) ***	
	Yes % (N)	No % (N)	Yes % (N)	No % (N)	Yes % (N)	No % (N)

Well informed	38 (121/317)	62 (196/317)	62 (122/198)	38 (76/198)	52 (103/198)	48 (95/198)
Moderately well informed	32 (163/509)	58 (346/509)	59 (145/245)	41(100/245)	41 (101/245)	59 (144/245)
Not at all informed	20 (39/193)	80 (154/193)	37 (23/62)	63 (39/62)	26 (16/62)	74 (46/62)
Total	323/1019^1^	696/1019^1^	290/505^1^	215/505^1^	220/505^1^	285/505^1^

* χ^2 ^= 17.92, p < .0001, ** χ^2 ^= 12.21, p < .002, *** χ^2 ^= 14.26, p < .001

^1 ^Total N = 1019 for entire sample, and 505 for those 40 and over due to missing values in well-informed variable

When asked about their three best sources of breast cancer information, women listed the print media (576) most frequently, followed by their health care providers (543). Other sources of breast cancer information were: television (204); one-on-one conversations with friends, parents, siblings, cosmetologists, and others (194); work/school (57); seminars/health programs (47); nurses and other health professionals (43); and the Internet (25). Total responses add up to more than 100% because women could provide more than one response to this question. These data were reported in an earlier paper that described the participants' perceptions of their most serious health threats [11].

Participant' knowledge of screening guidelines was evaluated by asking the participants to list the age at which women should start: 1) monthly BSE; 2) CBE every three years; and 3) annual mammogram. Only 31% (324/1055) of the women correctly reported that CBE every three years should be done between the ages of 20 and 39 years. For mammography, only 13% (140/1055) correctly reported that annual yearly mammography should be started at age 40. Only 8% (81/1055) of the women reported that monthly BSE screening should begin at age 20. (The most common answer (14% (146/1055)) for this question was puberty.)

### Attitudes toward illness

Even though women's screening rates were low, they were aware of the value of the screening process. Ninety-four percent (992/1055) disagreed with the statement, "I have a fairly fatalistic view when it comes to illness." Most (91% (957/1055)) agreed with the statement, "The more I know about a disease, the more control I have."

### Behaviors: adherence to screening guidelines

Adherence to screening guidelines was defined as having had a breast self-exam (BSE) within the past month, and having had a clinical breast exam (CBE) and mammogram in the past year for women 40 years and older [2]. Monthly BSEs were reported by 32% (321/1013) of the total sample. Among those 40 and older (n = 527), CBE in the past year was reported by 57% (298) and a mammogram in the past year was reported by 43% (226). Women's screening adherence by their age group is shown in Table 2. Adherence to BSE and mammography varied by age. BSE was reported more often by women between the ages of 40–59 compared to those who were in the youngest and oldest age groups. Self-report of mammograms in the past year were significantly higher among women 50–59 years compared to women 40–49 and those 60 years and older. Adherence to screening guidelines did not vary by educational level with the exception of CBE. Women who reported more education were also more likely to report screening using CBE (Table 3).

**Table 2:** Screening adherence for BSE, CBE, and mammogram by age group

Age group	Reported BSE within past month *		Reported CBE within past year (for women 40 + only)		Reported Mammogram within past year (for women 40 + only) **	
	Yes % (N)	No % (N)	Yes % (N)	No % (N)	Yes % (N)	No % (N)

20–39	28 (134/486)	72 (352/486)	N/A	N/A	N/A	N/A
40–49	39 (102/265)	61 (163/265)	60 (159/265)	40(106/265)	38 (100/265)	62 (165/265)
50–59	35 (49/139)	65 (90/139)	58 (80/139)	42 (59/139)	51 (71/139)	49 (68/139)
60+	29 (36/123)	71 (87/123)	48 (59/123)	52 (64/123)	45 (55/123)	55 (68/123)
Total	32 (321/1013)^1^	68 (692/1013)^1^	57 (298/527)^1^	43 (229/527)^1^	43 (226/527)^1^	57 (301/527)^1^

* χ^2 ^= 10.62, p = .014, ** χ^2 ^= 6.87, p = .033

^1 ^Total N = 1013 for entire sample, and 527 for those 40 and over due to missing values in age

**Table 3:** Screening adherence by education level

Education level	Reported BSE within past month		Reported CBE within past year (for women 40 + only) *		Reported Mammogram within past year (for women 40 + only)	
	Yes % (N)	No % (N)	Yes % (N)	No % (N)	Yes % (N)	No % (N)

High school graduate or less	25 (30/122)	75 (92/122)	44 (34/77)	56 (43/77)	38 (29/77)	62 (48/77)
Some college or vocational	31 (169/547)	69 (378/547)	59 (141/239)	41 (98/239)	46 (109/239)	54 (130/239)
College graduate or more	35 (123/356)	65 (233/356)	61 (122/199)	39 (77/199)	43 (85/199)	57 (114/199)
Total	322/1025 ^1^	703/1025 ^1^	297/515 ^1^	218/515 ^1^	223/515 ^1^	292/515 ^1^

* χ^2 ^= 7.01, p = .03

^1 ^Total N = 1025 for entire sample and 515 for those 40 and over due to missing values in educational level

### Perceptions of support

Less than half of the women (49% (514/1055)) reported that their closest family members and friends were very supportive in helping them to adopt ways to improve their health, 22% (237/1055) saying that their family and friends were mildly supportive, and 24% (253/1055) stating that their family and friends were not very supportive. Five percent (51/1055) did not respond.

### Perceived susceptibility

To estimate African American women's perceptions of their susceptibility to breast cancer and the severity of the disease in the African American community, they were asked to list "the four most serious health problems facing Black women." Breast cancer was mentioned as one of four serious health problems by 51% (541/1055) of the women [11]. Diabetes was mentioned by 59% (625/1055), and high blood pressure was mentioned by 53% (556/1055) of the women. Of the 1,055 participants, breast cancer was listed on the first reply line by 310 women, suggesting that this health threat was uppermost in the minds of nearly one-third of the women in this study.

### Perceived benefits

Responses from women in this sample indicated that they perceived benefit to taking action in detecting breast cancer early. Ninety-four percent (989/1055) of the women disagreed with the statement, "Breast cancer early detection does not make a difference."

### Cues to action

Women were asked whether they or other members of their social circle had breast cancer. Women who reported knowing someone with breast cancer were categorized as having personal exposure to breast cancer. Only 1% (13/1055) reported having breast cancer themselves, 8% (82/1055) reported having family members with breast cancer, 21% (227/1055) reported having friends with breast cancer, and the rest had either no exposure (65% (683/1055)) to the disease or did not answer (5% (50/1055)). Using that information, women were categorized as those with prior personal exposure to breast cancer (30%) or those without prior personal exposure to breast cancer (65%).

Chi-square analysis showed that exposure was not significantly related to women's education level, their perception of breast cancer as a health threat, or how well informed they were about breast cancer. Exposure was, however, significantly related to age. Younger women were less likely to have had prior personal exposure to breast cancer (χ^2 ^= 12.84, p < .0001; 68% of women under 50 reported no exposure versus 56% of women 50 years and older reported no exposure). There was also a significant relationship between prior personal exposure to breast cancer and practice of BSE and mammogram (Table 4). Women who reported personal exposure to breast cancer were more likely to have performed BSE in the past month or have had a mammogram in the past than women without personal exposure.

**Table 4:** Screening adherence by exposure to breast cancer

Education level	Reported BSE within past month *		Reported CBE within past year (for women 40 + only)		Reported Mammogram within past year (for women 40 + only) **	
	Yes % (N)	No % (N)	Yes % (N)	No % (N)	Yes % (N)	No % (N)

No exposure	28 (194/683)	72 (489/683)	54 (169/315)	46 (146/315)	39 (123/315)	61 (192/315)
Personal exposure	36 (134/372)	64 (238/372)	61 (129/212)	39 (83/212)	49 (103/212)	51 (109/212)
Total	328/1055^1^	727/1055^1^	298/527^1^	229/527^1^	226/527^1^	301/527^1^

* χ^2 ^= 6.52, p < .011, ** χ^2 ^= 4.71, p < .03

^1 ^Total N = 1013 for entire sample, and 527 for those 40 and over due to missing values in age

### Self-efficacy

In response to an open-ended question about how to reduce their risk of dying from breast cancer, women could list three possible answers (Table 5). Overall, BSE was mentioned by 53%, CBE by 41% and mammograms by 33% of the women. Women who had reported that they felt well informed about breast cancer were significantly more likely (p < .001, data not shown) to list regular BSE, regular CBE, annual mammogram, and early detection as ways to reduce the risk of dying from breast cancer than women who had reported that they did not feel well informed. In a separate analysis, when participants' responses on only the first of three reply lines were considered, 35% (370/1055) of women reported regular BSE, 24% (253/1055) reported CBE, and 10% (108/1055) reported mammography as the strategies that first came to their minds as effective ways to reduce the chance of dying from breast cancer.

**Table 5:** Responses when asked to list the 'Ways to reduce the risk of dying from breast cancer'

Ways to reduce risk of dying from breast cancer	% (N) *
Regular Breast Self-Exam	53 (556)
Regular Clinical Breast Exam	41 (436)
Annual Mammogram	33 (349)
Low fat diet	15 (157)
Early detection	11 (120)
Exercise	5 (57)

* Women were able to provide more than one answer

## Discussion

The American Cancer Society (ACS) has recommended annual clinical breast exams and mammograms for all women age 40 and older, and the ACSs California Annual Facts and Figures 2007 noted that only 39% of the women had reported an annual mammogram in 1987, a number that rose to 57% in 2004 [13]. In this study, only 43% (226) of the women in that age group reported having had a mammogram in the past year during the period of data collection (1998 to 1999). Only 57% of the study participants aged 40 and older reported having had a clinical breast exam (CBE) in the past year. This low screening rate is even more alarming in light of the fact that this study likely recruited a greater proportion of participants who were socioeconomically advantaged compared to the community at-large. The participants in the study all had the discretionary funds necessary to purchase their aesthetic services at state-licensed salons and thus, represented the more advantaged members of the community. If screening rates decrease in parallel with women's decreasing financial wherewithal, the screening rates reported in this study may actually overestimate the screening rates for the community at large. Thus, it would appear that lower-than-optimal rates of adhering to recommended screening guidelines continue to contribute to the greater incidence of detecting breast cancer in more advanced stages among African American women.

Breast cancer was listed as one of four serious health problem facing African American women by 51% (541/1055) of the women. Additionally, 29% of the women listed breast cancer first in their list of problems. This high awareness of breast cancer shows a heightened sensitivity to it as compared to heart disease, the most common cause of deaths in the African American community [14]. The heightened awareness of the severity of breast cancer is contrary to past studies which reported that African American women are not aware of the threat of breast cancer in their community [8,15,16]. One possible explanation for this finding is that during the interval since prior studies were done, the various breast cancer education programs and fund raising campaigns for breast cancer have helped heighten women's awareness of breast cancer as a health threat.

Although the women in this study appeared to perceive they were susceptible to breast cancer, their adherence to breast cancer screening guidelines was low. Women who had no personal, familial, or social exposure to breast cancer were also least likely to report having been screened. This underscores the importance of continued work in educating and engaging African American women in dialogues about the importance of breast cancer screening. Further, the women in this study did not demonstrate widespread knowledge of the details related to the recommended screening guidelines. For example, BSE, the least effective method of screening, was reported as a reliable screening method more often than the more effective methods of CBE and mammography.

Previous literature suggests that high motivation for health concerns will lead to an increase in health promoting behaviors [17]. In this study, the women reported perceived benefits for preventative care, as well as a low fatalistic view on life but did not report high rates of adherence to screening guidelines. They also did not report widespread support from family and friends for engagement in health promoting lifestyles, a factor that could undermine their self-directed health promotion activities. These observations suggest that social factors may be associated with participants' lack of engagement in health promoting activities.

Nearly all the women in the study believed that they could influence their health. However, few women perceived that they possessed adequate breast cancer knowledge, thereby providing further support for the importance of the randomized controlled trial of a beauty salon-based breast cancer education program to increase breast cancer knowledge, which was planned to follow this baseline data collection. Among those participants who reported that they possessed adequate breast cancer knowledge, there was a high association with adherence to recommended screening guidelines. Thus both the perceived and demonstrated lack of adequate breast cancer knowledge may help to explain the low screening rates reported by the participants in this study.

Recommendation by a health care provider is widely considered the most significant predictor of women's adherence to BC screening guidelines. Review of the literature suggests that African Americans view the medical establishment with mistrust and this may contribute to these sub-optimal screening rates [6,18]. In this study, however, health care professionals were the second most frequently listed among participants' best sources of breast cancer information, suggesting that the reported mistrust of the health community may not have been a significant barrier in increasing African American women's adherence to breast cancer screening guidelines. While health care providers have, and will continue to play, a key role in educating their patients about the need for screening and referring them for screening, they may need to be more proactive in encouraging their patients to engage in breast cancer screening activities, from engaging in advancing their patients' education to scheduling screening appointments before their patients leave the office.

In this study, women most frequently listed the media as their best source of breast cancer information. Since the public spends considerably more time exposed to the media, then to their health care providers, the media represents an important partner for the dissemination of health information. Thus these data also suggest that African American women will also benefit from partnerships between public health educators and media partners to speed the dissemination of breast cancer information and expand the messages' audience.

The study participants' awareness that breast cancer poses a serious health threat to African American women, suggests that the many messages about breast cancer that have been widely broadcast to women as well as the messages that have been specifically focused on African American (narrowcast), appear to have successfully reached African American women and have done so in a way that personalized the message. It is at this junction, in alignment with the Health Belief Model, that there seems to be room for improving women's knowledge about breast cancer so they can make well informed decisions, including those related to screening and treatment, as appropriate. The study which follows this baseline assessment will specifically explore whether giving African American women access to essential information about breast cancer, in a manner that makes it clear the information is intended specifically for African American women, will increase their screening rates.

## Limitations

The findings that evolved from this sample should be generalized with caution since they were developed based on a regional convenience sample. In addition, recruitment of these women from beauty salons may have led to a biased sample of women who are able to afford such services. As anticipated, the women who could afford to purchase beauty services at a neighborhood salon and would consent to research participation were also more likely to be better educated than the region's African American population at-large. This is a concern that plagues most research studies and must be taken into account when researchers consider the degree to which their findings may reasonably be generalized [11]. Also, for those who participated in the study, the very act of formally consenting to participate in a research study sets them apart from those women who would refuse such an invitation.

## Conclusion

Participants in this study reported low rates of adherence to recommended breast cancer screening guidelines. This study suggests that African American women are highly aware of the presence of breast cancer as a health concern and that they also perceive that breast cancer presents a serious health threat for African American women. Data from the African American women in this study suggest that an optimal intervention would address their need for information about when breast cancer screening should be started and which of the available screening methods will be the most effective methods for achieving increased rates of early breast cancer detection. Such an intervention was developed and tested via a randomized controlled trial. Those results will be reported in a subsequent manuscript.

## Competing interests

The author(s) declare that they have no competing interests.

## Authors' contributions

GRS designed and conducted the study and helped draft the manuscript. CK performed the statistical analysis and prepared the manuscript. JC, MC, and RW helped to collect data and prepare the manuscript. PW helped to prepare the manuscript. All authors read and approved the final manuscript.

## Pre-publication history

The pre-publication history for this paper can be accessed here:


